# Announcement

**DOI:** 10.1155/S1463924698000212

**Published:** 1998


					Journal of Automatic Chemistry, Vol. 20, No. 5 (September-October 1998) pp. 177
Announcement
International Ion Chromatography Symposium
Preliminary programme
The International Ion Chromatography Symposium
1998 will be held 28 September-1 October at the Osaka
Sun Palace Hotel in Osaka, Japan. Programme Chair-
man Kikuo Oikawa heads an organizing committee of
internationally renowned ion chromatography and ca-
pillary electrophoresis experts.
This year's programme features seven plenary lectures
and over 100 technical presentations. The enclosed
preliminary programme illustrates the diversity and
depth of the programme. Featured plenary speakers
include Hamish Small, the inventor of ion chromatogra-
phy, and several well-known scientists in this field of
separation science.
This three-day international meeting provides a unique
opportunity for scientists from a variety of disciplines to
exchange ideas and results on current methods and
procedures in ion chromatography and capillary electro-
phoresis for the separation and determination of small
organic and inorganic ions. Topic areas include:
IC separation selectivity and methodology
CE methods for ion analysis
Optimization/modelling
Advances in detection
Special sample treatment and preconcentration
procedures
Environmental applications
Pharmaceutical applications
Ion analysis in the semiconductor industry
Industrial applications
Novel applications
For more information, contact: Century International, Inc., P.O.
Box 493, Med]ield, MA 02052-0493, USA; Tel.: 0001 508/
359-8777; Fax: 0001 508/359-8778; e-mail: century@ixl.net
0142-0453/98 $12.00 1998 Taylor & Francis Ltd
177

				

